# The influence of online images on self-harm: A qualitative study of young people aged 16–24

**DOI:** 10.1016/j.adolescence.2017.08.001

**Published:** 2017-10

**Authors:** Nina Jacob, Rhiannon Evans, Jonathan Scourfield

**Affiliations:** a Cardiff School of Social Sciences, Cardiff University, The Glamorgan Building, King Edward VII Avenue, Cardiff CF10 3WT, UK; b DECIPHER, Cardiff School of Social Sciences, The Glamorgan Building, King Edward VII Avenue, Cardiff CF10 3WT, UK; c The Centre for Trials Research, Cardif University, Neuadd Meirionnydd, Heath Park, Cardiff CF14 4YS, UK

**Keywords:** Self-injury, Self-harm, Internet, Social media, Qualitative research

## Abstract

To date, research on the role of the Internet in self-harm has focused on young people's interaction via the medium of text, with limited consideration of the effect of images. This qualitative study explores how young people understand and use online images of self-harm. Semi-structured interviews were conducted with a community sample of 21 individuals aged 16–24 living in Wales, UK, with a previous history of self-harm. Interviewees reported the role of the Internet in normalising young people's self-harm. Images rather than textual interactions are the primary reason cited for using the Internet for self-harm purposes. Images invoke a physical reaction and inspire behavioural enactment, with Tumblr, which permits the sharing of images by anonymous individuals, being the preferred platform. Viewing online images serves a vital role in many young people's self-harm, as part of ritualistic practice. Online prevention and intervention need to attend to the importance of images.

## 1 Introduction

Self-harm is a major public health concern in relation to young people. It can be defined as a non-fatal act where an individual engages in a behaviour or ingests a substance with the intention of causing harm to themselves (Owen, Hansford, Sharkey, & Ford, 2016). The underlying suicidal intent associated with the behaviour has been contested, with increased differentiation between non-suicidal self-injury [NSSI] and acts with an associated suicide intent (Muehlenkamp and Kerr, 2010, Nock, 2001, Ougrin et al., 2012). However, both non-suicidal and suicidal self-harm share a range of risk factors (Mars et al., 2014), indicating location along the same continuum (Kapur, Cooper, O'Connor & Hawton, 2012). Community samples of non-clinical populations estimate the prevalence of self-harm in young people in the UK to range from 6.9% to 18.8% (Hawton et al., 2002, Kidger et al., 2012, Morey et al., 2008, O'Connor et al., 2008).

A number of risk factors associated with self-harm in young people have been identified (Hawton, Saunders, & O'Connor, 2012), with research increasingly attending to the role played by online spaces (Daine et al., 2013). Evidence indicates that 51.3% of young people who report self-harm have previously engaged in related Internet searches for self-harm or suicide related material (Mars et al., 2015). Positive influences associated with Internet use include the development of relationships with others, with connections offering empathy and support (Baker and Fortune, 2008, Lewis and Seko, 2016, Smithson et al., 2011). Online forums may reinforce positive behaviours and encourage help-seeking (Smithson et al., 2011). They have also been cited as a source of stress alleviation and coping (Harris and Roberts, 2013, Jones et al., 2011, Whitlock et al., 2006). Conversely, Internet use may confer harm due to the normalisation of self-harming behaviours, the sharing of practices and techniques, and the encouragement of concealment (Dunlop et al., 2011, Eichenberg, 2008, Lewis and Seko, 2016, Smithson et al., 2011). It may promote self-harm enactment, with a community study by O'Connor, Rasmussen, and Hawton (2014) reporting that 18% of secondary school-aged students were influenced to self-harm by social networking sites.

Despite increased empirical consideration of the prevalence of Internet use, and the mechanisms through which it influences self-harm in young people, the evidence-base remains limited. Firstly, existing research is predominantly characterised by online surveys or content analysis of community forums (Eichenberg, 2008, Harris et al., 2009, Mitchell and Ybarra, 2007, Whitlock et al., 2006), with only a small number of qualitative studies exploring the meanings young people attach to the Internet (Baker & Fortune, 2008). Secondly, despite emerging evidence reporting on the influence of the range of content hosted by various online media platforms (Harris & Roberts, 2013), there is a lack of research addressing how young people respond to different mediums of communication within these platforms. Notably, research has focused on the textual interaction of individuals, paying scant attention to imagery. In the small number of studies that have explored user-generated images, these have been said to reduce loneliness, mitigate self-harm enactment, and offer a means to support others (Baker and Lewis, 2013, Seko et al., 2015). Equally, they have been reported as a behavioural trigger (Baker & Lewis, 2013). Thirdly, there is a paucity of research on the online sites and platforms utilised as part of self-harming practices, and the perceived strengths and limitations associated with different online spaces (Lewis & Michal, 2016). The present study further progresses research on the influence of the Internet on young people's self-harming behaviours, reporting qualitative data exploring the distinct appeal of different media of communication, notably the visual medium, and the preferred online platforms that support the use of imagery.

## 2 Methods

Participants were purposively sampled for the study. The inclusion criteria were: young people aged 16–25; and previous experience of self-harm. The definition of self-harm was not determined a priori, but conceptualised by the participant. Recruitment was conducted through the social networking site Facebook. This website was utilised as the study aimed to elicit the lived experiences of a diverse range of individuals, including those who were not proactively engaged in online self-harm communities. Adverts were deployed to the Facebook pages of individuals aged 16–25, listed as living in Wales, UK, and who presented a specific set of interests or ‘likes’. Individuals who had ‘liked’ pages relating to the improvement of wellbeing and mental health, specifically charities and youth groups based in Wales, were targeted alongside those who had also listed ‘self-harm’ and ‘suicide’ as an interest. Groups assembled around particular music subcultures (‘Goth’ and ‘emo’) were also targeted due to established associations with self-harm (Whitlock et al., 2006, Young et al., 2006). An advert was posted on the pages of identified Facebook accounts for a period of four weeks, with the advert containing brief information on the research study and a link to the study website.

A total of 41,988 accounts displayed the advert, with 744 people (1.8%) clicking through to the main study website. Of these individuals, 49 (6.6%) indicated interest in study participation by leaving their contact details. This response is comparable to other studies that have utilised Facebook for recruitment (Fenner et al., 2012). Whilst the inclusion criteria was up to age 25, twenty one individuals aged 16–24 participated in the study. The mean age was 19, three were male and 18 were female. The mean age for commencement of self-harm behaviours was thirteen. Sixteen participants (76%) had sought professional help for their self-harm and 8 (38%) had presented to the Accident and Emergency department for their injuries. The vast majority of the sample engaged in cutting as the principle means of injury, although a range of behaviours were described including burning, bone breaking, skin picking and overdosing. That said, these practices were not static, with behaviours often presented as evolving and contradictory. Of the remaining twenty-eight individuals who registered interest, communication could not be established with ten young people and communication ended with the remaining eighteen before an interview could be arranged. In most cases, despite initially indicating interest in participating in the study, all further contact was not responded to. Subsequent contact from the researcher was limited to three attempts. In two cases, potential participants misjudged their required involvement, assuming it was an online survey and so declined participation. One further potential participant did not turn up to an arranged interview and did not respond to subsequent contact. Demographics for this group were similar to those recruited; two male and 16 female. In all cases, the researcher expressed appreciation for their interest via email, and also signposted on to relevant services.

Semi-structured interviews were undertaken. Nineteen were conducted individually, and one was conducted in a dyad. Interviews were led by the primary researcher [NJ]. The interview setting was decided through discussion between the researcher and participant. Interviews were conducted at a university, library, or café. The interview topic guide explored young people's lived experiences of self-harm, notably: motivations for self-harm; receipt of formal and informal support; use of the Internet; navigation of the Internet prior to, during, and following engagement in self-harm; perceptions and experiences of different online content and mediums of communication; and the interaction of online behaviour with real world behaviour. Data were recorded using a digital audio recording device and transcribed verbatim by a professional transcription service. Ethical approval for this study was provided by Cardiff University School of Social Sciences Research Ethics Committee.

Thematic analysis was conducted. Textual data were repeatedly read and coded inductively, with open coding being employed. A coding frame was developed by the primary researcher [NJ] and verified by a second member of the research team [RE]. Codes were subsequently applied to the whole corpus of data. A constant comparative approach was used to identify similarities and differences between accounts, with initial analyses of early interviews used to highlight gaps in knowledge associated with varying levels of engagement with the Internet. For instance, whilst analysis commenced with the broad focus of Internet use, as it progressed nuances within participant's experiences and perspectives emerged, particularly with regards to the degree of passive or active engagement online. Analysis was conducted by the primary researcher [NJ] with the remainder of the research team verifying the construct of categories and data interpretation [RE/JS]. Discrepancies in interpretation were resolved through discussion. Data analysis and data generation were conducted iteratively, with emerging themes being used to refine and further develop the topic guide. For instance, the centrality of images quickly became apparent early in data collection and consequently was explored in greater detail during subsequent interviews. NVivo version 10 supported data storage and analysis.

## 3 Results

Results are presented in three sections. First, they address the motivations for participants' use of the Internet as part of their self-harming practices. Second, they explore the influence of online imagery, including the impact of exposure to these images. Third, they consider the distinct appeal of different social media platforms for young people engaging in self-harm.

### 3.1 The role of the internet in young people's self-harm

Participants acknowledged the role of the Internet in their self-harm practices. Some cited it as a catalyst in the commencement of these behaviours, where individuals had researched advice or support for emotional distress, but self-harm had ‘*just come up*’ during the search with the retrieval of instructions and images. However, for the most part, self-harm was not an unknown act inadvertently discovered; it was already a part of young people's lives. Many initially engaged with the Internet with a generic search for self-harm in the attempt to make sense of their behaviours, before discovering and being drawn into online communities.

Use of the Internet was perceived to have a discernible impact on participants' self-harm, primarily through the reinforcement or encouragement of behavioural enactment. For a number of individuals, online community exchanges had supported the normalisation of harm, leading to its construction as a routine, everyday activity:

*I was in complete secrecy about my self-harm, but then I'd go home and I had all these people on the computer who I could talk to, who would support me, who didn't see self-harm as some weird thing that was a massive problem. They saw, you know, they saw it the same as making a cup of tea in the morning when you wake up, it was completely fine. But the problem with that was my cuts went from scratches to a lot deeper. (I20, female, aged 19)*

Online content and communities also afforded opportunities to discover and share practices and techniques:

*I would just look at tips, how to hide it you know how to make it, make it hurt more and things like that, and yeah just kind of, I don't know, I don't know. I did go on there for a positive reason in the beginning, but after that I was just looking at negatives so like pro self-harm sites and that was it then.* (I13, female, aged 23)

*I was thinking if I can do this one way, how can I do it other ways, so then I was thinking, um … Most of it comes from online, because I spend most of my time on my laptop or on the computer anyway, and erm I was looking for something with no visual scars or anything like that, you just felt completely out of your body, and that you just didn't feel nothing whatsoever, I was looking for that. So then I found people saying “oh yeah sometimes I take drugs because it makes me feel out of my body”.* (I15, male, aged 17)

*Because I saw on the Internet wrist banging, I'd heard the term before, wanted to know what it was and then I was like, oh I'll give that a try”* (I2, female, aged 17)

Access to means was a notable feature of online interactions, with the Internet further supporting procurement by permitting young people to circumvent ‘real world’ restrictions placed on the purchase of self-harming ‘tools’ on account of their age:

*I used to buy razor blades on eBay because they come in the post, you know, no-one would know what they were, um, because I thought oh god, I'm only like 15, 16, I can't go in to a shop and buy all these blades, you know, so I used eBay*. (I6, female, aged 24)

Through the increase in knowledge about the techniques of self-harm, some participants discussed the amplification and severity of their behaviour as they utilised more injurious methods. Equally, they became motivated to engage in further harm through investment in the notion of ‘not good enough’, where exposure to other individuals' severe acts made them want to become better self-harmers:

*I'd look for different methods of self-harm, salt and ice burns, bone breaking, and things like that and then I'd share what I did … Once my self-harm escalated it stayed quite heightened, like I couldn't just make one little cut, it just was impossible. I'd just look at myself in the mirror and laugh, be like, “Get a grip. What are you doing? That's not nearly good enough.* (I20, female, aged 19)

### 3.2 The influence of online imagery on young people's self-harm

While participants cited the importance of textual interaction within online communities, they also considered the move away from the privileging of such discursively driven spaces towards the production and consumption of images. Indeed, nearly three quarters indicated that imagery (notably photographs) were the primary reason for their utilisation of the Internet, due to a powerful and physical reaction that triggered the desire to harm:

*I would go on Google and look at the images; because like I've said I get a really fast heart rate and its triggering and it's like a rush and it became this like addictive high and then each picture had to be worse than the one before*. (I1, female, aged 22)

*The quotes wouldn't, but the pictures would cos the quotes it's just like reading, I can't visually see it whereas when you visually see it you kind of wanna do it then.* (I11, female, aged 18)

The power of the image primarily centred on their ability to *‘bring back memories’* of previous self-harming episodes or the ease with which they allowed the individual to envisage how others experience the act:

*But it's not like I saw a picture and then automatically thought ‘Oh, I must self-harm’ … it would be like a building thing, like if I saw a particular picture, I don't know, if someone had done like a nice juicy bruise or something, I'd be like oh, I can imagine what that feels like, and even the thought of it kind of makes me feel a bit better. Like makes me feel like I want one or something like that.* (I9, female, aged 20)

Participants also spoke of being inspired to recreate certain sets of practices presented by particular images:

*I remember I did think once like oh, um, yeah I'm going to … I'm going to try that next time to see, you know, if I go deeper will I get a better effect. It wasn't so much oh I want to have deeper cuts than them more that I needed to do what they had done*. (I6, female, aged 24)

Discussion was further characterised by a sense of competition, with individuals desiring to emulate the depicted harm whilst chiding themselves when they failed to engage with more sophisticated and severe techniques:

*Why can't I do it like that? … Because when I first started to do it, it was like with bits of can and bits of tin and then I started to bite the blades out of razors and just like using them like they did.* (I13, female, aged 23)

For many, reliance on images as a trigger had led to them assuming a vital role within their ritualistic practice, with ‘sessions’ often commencing with the retrieval of an online image:

*I realised that I was looking at them so that I could actually do it. I'd look first, then do. I'd look at it and I'd be like, ‘That's what I need to do’. And I'd mark with pens on my arms and my legs; it's got to be that wide. If it's not that wide you don't stop and I'd be cutting until I was absolutely exhausted.* (I20, female, aged 19)

*If I want to harm and I don't think I'll do it bad enough, I go on to, I go online and then I look for triggers and then when I'm triggering I know I will do it worse than what I would have done before.* (I14, female, aged 18)

There was some indication that individuals who shared their own images of injuries engaged in more extreme self-harm behaviours. The small minority who did upload their own pictures not only described severe injuries, there was also a sense that they were encouraged by the wider online self-harm community, thus potentially driving forward severe self-harm.

*INT: So why did you … at the point that you started posting them on there why … like what was going through your mind to make you want to do that?*

*RES: Maybe I'd get sympathy maybe in a weird of kind of way … but they were like oh but it wasn't real it was like, yeah. And I started getting … started self-harming more.* (I2, female, aged 17)

*There's a picture on there that I uploaded and it was of my self-harm, but it wasn't for attention, it was just for expression and then I got loads of shit for it, that it wasn't deep enough.* (I4, female, aged 16)

That said, this finding should be treated with caution as some extreme behaviours were also found amongst participants who only viewed images created by others and did not upload their own. Interviewees who were overtly critical of the sharing of self-harm images appeared to engage in a less severe form of self-harm. Amongst this group the rejection of public displays of self-harm were based upon the belief that it should be a private activity:

*It's like; self-harm is like a kind of an intimate experience with yourself. Um, you know, you don't want to share it with the world, you know? You get other people that do it and just put it all over Facebook and Tumblr and stuff. But I'm not the type of person.* (I3, male, aged 16)

There was further concern expressed that the posting of images would be interpreted as attention-seeking, a category of behaviour to be avoided:

*I didn't think it was shameful or anything like that, I just thought … I think I thought that if people saw it, it would be attention-seeking and I hate that.* (I12, female, aged 22)

Equally, the online display was seen as being the province of the Goth and emo, and participants were inclined to distance themselves from this sub-cultural identity, and so avoided the posting of images of their behaviour in case they were assigned this identity by the online community.

### 3.3 The use of online media platforms for displaying self-harm imagery

The virtual world is in a constant state of flux with websites regularly being created and deleted. Self-harm sites are no different. It has been noted that the once prevalent self-harm forums, popular with users and researchers alike, are no longer utilised to such an extent (Harris & Roberts, 2013). Participants spoke of the challenges of finding these online communities due to invisibility and inactivity, with some reminiscing about some online forums that had lapsed:

*I'm kind of sad that Psyke is not very active, I mean last time I went on their site it was like last updated 2009 or something. Ages ago.* (I9, female, aged 20)

Whilst there has been a propensity within research on self-harm to classify social media and microblogging into one homogeneous category, participants delineated the nuances between different platforms and discussed the distinct appeal of particular sites that had come to replace the older forums.

Tumblr was frequently cited as a preferential online space and was often discussed as synonymous with online self-harm activity; *‘all self-harm links to Tumblr mostly’.* There were a number of features associated with Tumblr that participants appreciated.

First, identification of relevant blogs and content are facilitated through the simple searching of keywords and tags, which allows for communities to easily assemble around self-harm. Second, it is amenable to the sharing of images. Tumblr is predominantly used as a photo-blogging platform (Duffy, 2013), with half of all posts being photographs (Marquart, 2010). Participants stated that they used the platform so that they could share self-harm pictures.

Third, it is perceived to permit an immediate and intimate connection with others, and is not encumbered by the monitoring and intervention provided by other social media and microblogging sties:

*So I wrote in like pro self-harm. Nothing came up. Self-harm, nothing came up and like I could look on pictures of self-harm but there was just nothing, it was all, you know, monitored support websites and stuff … so I thought well this isn't working and I searched it on Tumblr and then I found a hell of a lot like oh my life's over.* (I2, female, aged 17)

*I can't remember how I found it, but you type in cutting and then everything comes up, all links, most of the things that come up are linked to Tumblr. So you type in self-harm … And then people share like pictures of self-harm, which is normal, it's Tumblr, that's what people use it for really.* (I19, male, aged 16)

The lack of scrutiny and moderation, where you can purportedly *“do what the hell you like”,* together with perceived anonymity, meant that the site was considered to be more authentic than alternative platforms. However, the freedom of access to Tumblr, combined with user discretion to share the most severe and stark images of self-harm, has led to the normalisation and exacerbation of self-harm for participants:

*Kids as young as 12 can use it and anybody can look at your blog, nothing is really private and there's a big self-harm community on there that you can get sucked into and I got sucked into it and it did sort of increase the intensity of my self-harm again. Like it went from sort of little gashes in my legs to I just … I wasn't happy until I could see an artery and I'd cut through it* (I20, female, aged 19).

## 4 Discussion

The present study aimed to progress existing research on young people's use of the Internet for self-harm purposes through consideration of the distinct appeal of different sites within these online spaces and the various platforms that support individuals' needs. Although there has been extensive documentation of the positive and negative impacts associated with utilisation of the Internet (Baker and Fortune, 2008, Dunlop et al., 2011, Eichenberg, 2008, Harris and Roberts, 2013, Lewis and Seko, 2016, Smithson et al., 2011, Whitlock et al., 2006), including an increased focus on the influence of photographs or computer-generated images (Baker and Lewis, 2013, Lewis and Michal, 2016, Seko et al., 2015), there remains limited empirical exploration of *how* individuals interact with imagery as part of their self-harm practices.

While some individuals reported discovery of self-harm through Internet searching for a tangential topic, which led to commencement of the behaviour, the majority engaged with online spaces to support and further develop a pre-existing set of self-harming practices. In alignment with existing research on the role of the Internet in behavioural enactment, engagement with online communities often led to an exacerbation of self-harm due to normalisation and increased exposure and access to new techniques (Harris and Roberts, 2013, Jones et al., 2011, Whitlock et al., 2006). That said, it should be noted that other research has found a dichotomy in response to the consumption of self-harm imagery, with positive responses including decreased frequency of injuries and increased feelings of support being reported (Baker & Lewis, 2013). Reasons behind positive associations include images acting as a substitute for engaging in the behaviour (Baker & Lewis, 2013) whereby uploading content acts as a vehicle for an emotional outlet, quelling the urge to harm (Seko et al., 2015).

Despite the use of the Internet, it is apparent that not all platforms and micro-blogging sites are endorsed as equally conducive to sharing self-harm related materials or engagement in online interactions. Such a finding challenges the failure of researchers in this field to differentiate the corpus of online spaces, focusing only on the distinction between online and offline self-harm activity (Lewis & Michal, 2016). Although some individuals in the study lamented the lapsing of previously popular forums, the primacy of Tumblr in supporting young people's self-harming needs was clearly evident.

Reasons for preferring this platform included the simplistic nature of its functionality and the anonymity and privacy afforded users. These notions do appear somewhat problematic, given that the premise of Tumblr is to be an open and visible platform. Yet as Livingstone (2008) suggests, young people operate within a highly nuanced and precise understanding of privacy, one which sees it in terms of limits to self-expression. Consequently, privacy is no longer about the actual disclosure of information, rather about having control over who knows what about you.

For many research participants, the predominant reason for use of Tumblr is its privileging of the image. Indeed, online communities seem to be increasingly assembled around the production and sharing of imagery in an attempt to document their self-harm journey. The purposeful searching of images may be bound up with the changing ways in which individuals use online media more broadly, where the publishing of photographs documenting everyday life is a common practice amongst young people. As Sternudd (2012) comments, the sharing of photographs is such a widespread online activity that it would be more surprising if self-harm was not a part of the practice.

The power of the image as part of the ritualistic practice of self-harm was evident throughout participants' accounts. Indeed it has emerged as a vital element of the behaviour, serving as essential precursor to commencement of the act (Baker & Lewis, 2013). The importance assigned to images is reflected in young people's tendency to couch discussions in terms of ‘addiction’ or compulsion. Psychological studies have understood self-harm as most likely to occur when an individual cannot control their impulses, in addition to a need to escape or avoid an effective difficult situation or emotion (Chapman, Gratz, & Brown, 2006).

Stark and severe images have the capacity to induce a physical reaction that invokes a strong desire to engage in harm. Previous research has highlighted that the visual aspect of self-harm, from dripping blood to the “nice juicy bruise” are as much a part of the act as the physical hurt caused (Sternudd, 2014). As such, images might fulfil part of this function through the provision of important visual stimuli. They equally reflect some of the relational aspects of self-harm, and the interactions of online communities of self-harmers. Individuals spoke of being inspired to enact the practices of others, whilst simultaneously chastising themselves if they did not engage in the same extent of behaviour.

Whilst the impact of imagery has been previously underexplored and underestimated, we must juxtapose the position of young people who deem it necessary to their self-harm with those who actively resist online visual displays. Rejection of the public display of self-harm online was based upon the belief that it should be a private activity and that publishing images would be viewed as attention-seeking. Other research has found that for some young people, public displays of self-harm undermine the credibility of the act, with such behaviour considered less authentic than keeping injuries private (Scourfield, Roen, & McDermott, 2011). There was also resistance to the display of images from participants who felt they perpetuated stereotypes of self-harm, conflating it with Goth and emo subcultures. Of note, these individuals had an individualised discourse of their own self-harm journeys and practices, grounded in their agency and choice, rather than the norms prescribed by a group identity.

Population-level and targeted online prevention and intervention approaches for self-harm remain limited (Jacob, Evans and Scourfield, 2014). As research progresses, intervention development should focus on the place of imagery, whilst also being sensitive to young people's preferred media. Furthermore, given the ever-evolving nature of young people's use of the Internet for self-harm related activity, particularly with regards to the changing preferences for platforms and micro-blogging sites, mixed –methods research needs to continue to understand how individuals and communities interact with online spaces, and the impact these interactions have on self-harm.

## 5 Limitations

The study was limited by its sampling procedures, and participants may not have been representative of young people who self-harm and utilise the Internet. Recruitment was restricted to individuals who hold a Facebook account, and given the reported gendered uptake of this social media site coupled with the known gendered nature of self-harm it is important to be aware of the potential bias this sample has towards the experiences of young women (Thompson & Lougheed, 2012). Furthermore, the posting of study adverts on the pages of individuals a priori hypothesised to have a higher prevalence rate of self-harm (e.g. musical sub-cultures), may have led to the exclusion of other socio-demographic profiles that would provide a different sets of encounters with the Internet and online imagery. There may equally have been a selection bias in relation to individuals responding to the advert and proceeding to participate in an interview, with women more likely to volunteer to take part in studies (Porter & Whitcomb, 2005). That said, over half of those who initially responded did not take part in the study, an occurrence that has the potential for bias given that we have limited data on this group. The final sample included a higher proportion of females with only three males included in the study. However, there was no discernible difference across the sample in relation to gender. Of the three male participants, two used the internet for self-harm and actively consumed images for this purpose. Of the eighteen females, 11 sought out pictures of self-harm, a further three shared their own images and the remaining four were somewhat resistant to this behaviour. Despite these limitations, the sampling and recruitment procedures permitted the study to go beyond the samples included in the majority of studies considering the role of the Internet in young people's self-harm and suicide, where recruitment is conducted through established online community groups.

## 6 Conclusion

The Internet plays a vital role in young people's self-harm practices, with the present study supporting the negative aspects of this impact, notably through normalisation of behavioural enactment and sharing of techniques. The study has progressed understanding of how young people use online spaces and mediums through exploration of the primacy of images. In particular, images are sought out due to their capacity to invoke a physical reaction that inspires a desire to harm. The primacy of the Tumblr platform for engaging in online self-harm related activities is partly a result of its privileging of the photographic documentation of individuals' lives. Further research needs to continue to move beyond the homogenisation of online spaces and disentangle young people's interaction with different mediums across different platforms. Effective online prevention and intervention approaches for self-harm remains limited, and future development should more closely attend to the complex and ever-evolving interaction of individuals with online spaces.

## Conflicts of interest

The authors have no conflicts of interest.
